# Oxysterol-binding protein-1 (OSBP1) modulates processing and trafficking of the amyloid precursor protein

**DOI:** 10.1186/1750-1326-3-5

**Published:** 2008-03-18

**Authors:** Celina V Zerbinatti, Joanna M Cordy, Ci-Di Chen, Maria Guillily, Sokreine Suon, William J Ray, Guy R Seabrook, Carmela R Abraham, Benjamin Wolozin

**Affiliations:** 1 Department of Alzheimer's Research, Merck & Co., Inc., West Point, PA, 19486, USA; 2 Department of Pharmacology, Boston University School of Medicine, Boston, MA, 02118, USA; 3 Department of Biochemistry, Boston University School of Medicine, Boston, MA, 02118, USA

## Abstract

**Background:**

Evidence from biochemical, epidemiological and genetic findings indicates that cholesterol levels are linked to amyloid-β (Aβ) production and Alzheimer's disease (AD). Oxysterols, which are cholesterol-derived ligands of the liver X receptors (LXRs) and oxysterol binding proteins, strongly regulate the processing of amyloid precursor protein (APP). Although LXRs have been studied extensively, little is known about the biology of oxysterol binding proteins. Oxysterol-binding protein 1 (OSBP1) is a member of a family of sterol-binding proteins with roles in lipid metabolism, regulation of secretory vesicle generation and signal transduction, and it is thought that these proteins may act as sterol sensors to control a variety of sterol-dependent cellular processes.

**Results:**

We investigated whether OSBP1 was involved in regulating APP processing and found that overexpression of OSBP1 downregulated the amyloidogenic processing of APP, while OSBP1 knockdown had the opposite effect. In addition, we found that OSBP1 altered the trafficking of APP-Notch2 dimers by causing their accumulation in the Golgi, an effect that could be reversed by treating cells with OSBP1 ligand, 25-hydroxycholesterol.

**Conclusion:**

These results suggest that OSBP1 could play a role in linking cholesterol metabolism with intracellular APP trafficking and Aβ production, and more importantly indicate that OSBP1 could provide an alternative target for Aβ-directed therapeutic.

## Background

One of the major pathological hallmarks of AD is the deposition of extracellular plaques composed predominantly of the 4 kDa amyloid-β peptide (Aβ) [1,2]. This peptide is derived from proteolytic processing of the amyloid precursor protein (APP) by two distinct enzymatic activities, β- and γ-secretases. A third enzyme, named α-secretase, is also able to cleave APP but within the Aβ region, precluding Aβ formation. Several different α-secretases have been identified, including ADAM-9, -10 and ADAM-17/TACE [3-5]. Cleavage of APP by α-secretase has both a constitutive and a regulated component. The regulated component is mediated by TACE and is stimulated by both protein kinase C (PKC) [6] and extracellular signal-regulated kinase (ERK) [7]. The β-cleavage of APP occurs via the action of BACE1, a membrane-bound aspartyl protease primarily localized to the Golgi and endosomes [8,9]. Following α- or β-cleavage, the C-terminal fragment of APP (APP-CTFα/β) is subsequently cleaved by γ-secretase, which exists mainly in the Golgi and endoplasmic reticulum (ER) and is a complex made up of four proteins: presenilin, nicastrin, Aph-1 and Pen2 [10]. γ-secretase also has many other substrates, including the cell surface receptor Notch and its homologue Notch2 [11,12].

APP processing by the secretases is modulated by many factors including oxidative stress, ceramide, NSAIDs, sphingolipids and cholesterol [13-16]. Increasing evidence suggests that the metabolism of cholesterol, APP and Aβ are strongly interdependent. Many studies show that decreasing cellular cholesterol disrupts lipid rafts and interferes with APP processing [17], and recent studies have shown that Aβ, APP and presenilin can also regulate production of cholesterol and sphingomyelin [18]. Oxysterols, products of cholesterol oxidation, have also been shown to reduce Aβ secretion [19,20] and therefore could provide a potential link between cellular cholesterol levels and Aβ secretion.

Oxysterol-binding protein-1 (OSBP1) is the original member of a family of lipid-binding proteins, which consists of 12 members in humans. The other members of the family are referred to as OSBP-related proteins (ORPs) [21]. Most of the ORPs have a plextrin homology (PH) domain at the N-terminal end, while all have a ligand binding domain (LBD) at the C-terminal end [21]. In the case of OSBP1, the LBD binds cholesterol and its catabolic derivative, 25-hydroxycholesterol (25OH) [22,23]. Many other ORPs also bind 25OH [24], while others have been shown to bind different lipids including phosphoinositides and phosphatidic acid [25,26]. Most ORPs localize mainly to the cytosol, but association with membrane organelles can occur and is regulated by lipid binding to the LBD. Several ORPs also present alternatively spliced variants [21].

The LBD of OSBP1 contains two separate binding sites, one for cholesterol and one for 25OH, which enables OSBP1 to regulate the extracellular ERK signalling pathway [22]. When bound to cholesterol alone, OSBP1 exists in the cytoplasm and ER, where it also binds two phosphatases, the serine/threonine phosphatase PP2A and a PTPPBS class tyrosine phosphatase. Binding of these two phosphatases reduces their availability in the cytoplasm/ER and leads to increased activation of ERK [22]. Binding of 25OH to OSBP1 or removal of cholesterol leads to translocation to the Golgi apparatus, release of the phosphatases and reduced ERK activity.

The functions of OSBP1 and ORPs appear to be diverse, with potential roles including control of lipid metabolism, regulation of secretory vesicle generation and control of signaling pathways [27,28]. Recently it has been suggested that ORPs' main role is to act as sterol sensors and therefore control a wide variety of sterol-dependent cellular processes [29]. Consistent with the high cholesterol content of the CNS, OSBP1 and many ORPs are highly expressed in several brain regions [30,31], and we recently demonstrated that oxysterols can inhibit APP processing [32]. In the present study we investigated whether OSBP1 itself has a role in APP processing. We found that cellular levels of OSBP1 modulated the amyloidogenic processing of APP and altered the subcellular localization of APP-Notch2 heterodimers.

## Results

### Overexpression of OSBP1 inhibited APP processing and sAPP secretion

**Figure 1:**
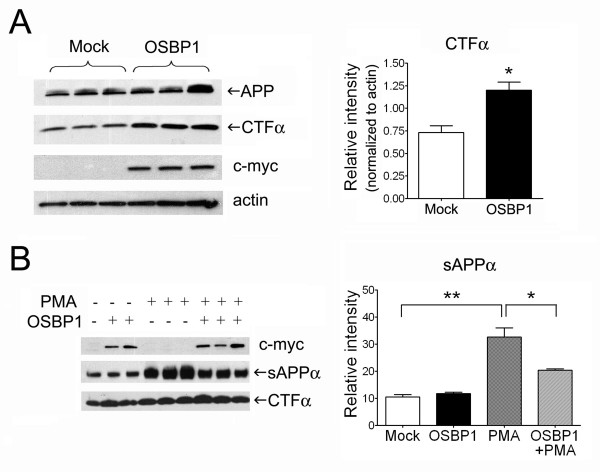
**OSBP1 overexpression increased the steady-state levels of α-secretase cleaved C-terminal fragment of APP (CTFα).A**, H4 cells stably overexpressing APP (H4-APP) were transfected with OSBP1 cDNA as described in Methods. Cell lysates from untransfected cells and those transiently overexpressing OSBP1 were immunoblotted with antibodies to the C-terminus of APP (upper panel), c-myc (middle panel), which detects the myc-tagged OSBP1, and actin, used for loading control. **B**, OSBP1 overexpression decreased PMA-regulated sAPPα secreted levels. Cells were treated with PMA as described in Methods. Proteins from cell lysates and media were separated by SDS-PAGE and lysates were immunoblotted with anti-myc antibody (upper panel). Media samples were analyzed for sAPPα using the 6E10 antibody (middle panel). Cell lysates were also analyzed for CTFα using an antibody to the C-terminus of APP (lower panel). Densitometric analysis of Western blots is shown on the right. *p < 0.05 and **p < 0.01 by Student's *t *test.

To determine the effects of OSBP1 overexpression on APP processing, H4 neuroglioma cells overexpressing wildtype APP (H4-APP) were transiently transfected with myc-tagged OSBP1. APP processing was evaluated by measuring the steady-state levels of APP C-terminal fragments (APP-CTFα/β) and soluble secreted APP species (sAPPα/β) that are produced following α- and β-cleavage, respectively. Overexpressing OSBP1 in H4-APP cells increased APP-CTFα levels by ~65% (Figure 1A). Since the expression level of full-length APP was not significantly altered by OSBP1 overexpression, increased APP-CTFα levels were likely a result of OSBP1-mediated modulation of APP processing. Interestingly, we found that OSBP1 overexpression did not alter constitutive sAPPα secretion, and it unexpectedly hampered the increase in secreted sAPPα levels when the regulated α-cleavage pathway was induced by PMA treatment (Figure 1B, middle panel). In addition, OSBP1 overexpression was unable to further alter the concomitant increase in APP-CTFα levels induced by PMA treatment alone (Figure 1B, lower panel). These results suggest that OSBP1 may modulate APP processing via complex mechanisms, perhaps by affecting both the non-amyloidogenic trafficking of APP and the clearance of sAPPα.

**Figure 2:**
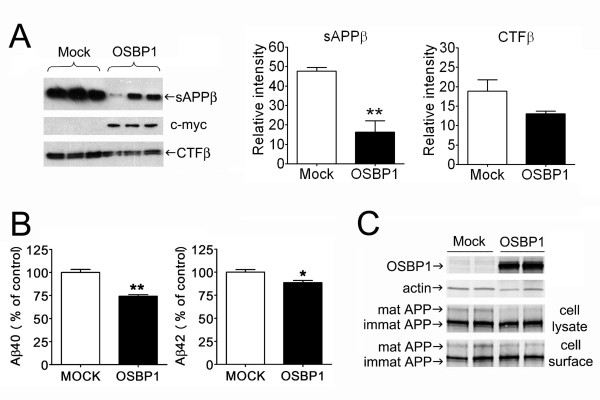
**OSBP1 overexpression inhibited β-secretase cleaved APP-CTF (CTFβ) and decreased sAPPβ, Aβ40, and Aβ42 secreted levels, without affecting total or cell surface steady-state levels of full-length APP.A**, H4-APP cells were transfected with OSBP1. After 48 h, medium was removed and replaced with fresh optiMEM. Following 3 h incubation, media were collected and concentrated using Vivaspin columns. Proteins from lysates and media were separated by SDS-PAGE and media samples were immunoblotted using the 192 wt antibody, against the β-cleaved epitope of APP (top panel); lysates were blotted with anti c-myc antibody to confirm OSBP1 expression (middle panel) and with 6E10 antibody, which recognizes APP-CTFβ (lower panel). Densitometric analysis is shown on the right. **B**, Secreted Aβ40 and Aβ42 levels in 24 h conditioned media from mock transfected and OSBP1-overexpressing HEK-APPNFEV cells were measured by ELISA. **C**, HEK-APPNFEV cells were transfected with OSBP1 cDNA as described in Methods. After 48 h incubation, cell monolayers were biotinylated prior to lysis. Total and cell-surface biotinylated proteins were separated and analyzed by Western blotting using antibodies to OSBP1, APP and actin as loading control. *p < 0.05 and **p < 0.01 by Student's *t *test.

Processing of APP via the amyloidogenic pathway was found to be down-regulated by increased OSBP1 levels. In contrast to the unpredictable changes observed within the α-cleaved APP metabolites, all β-cleaved metabolites were consistently altered. A 30% decrease in cellular levels of APP-CTFβ and secreted sAPPβ were observed with OSBP1 overexpression in H4-APP cells (Figure 2A). The effect of OSBP1 overexpression on Aβ40 and Aβ42 secretion was investigated in HEK cells stably expressing APPNFEV, a mutated human APP exhibiting 100-fold enhanced BACE1 cleavage rate relative to the APP wild type substrate [33], therefore allowing for Aβ detection in the media by ELISA. As expected, secreted Aβ40 and Aβ42 levels were significantly reduced in HEK-APPNEFV cells expressing OSBP1 (Figure 2B). Similar to the findings in H4-APP cells, OSBP1 overexpression in HEK-APPNFEV cells did not produce significant changes in the steady-state levels of total APP nor led to changes in cell surface distribution of APP as assessed by cell surface biotinylation experiments (Figure 2C). Levels of sAPPα in the media measured by ELISA in HEK-APPNFEV were also unexpectedly reduced by 24% in HEK-APPNEFV overexpressing OSBP1 (data not shown), suggesting again that OSBP1 overexpression impacts the non-amyloidogenic processing of APP in a more complex manner.

### Knockdown of OSBP1 increased β-site APP processing and Aβ secretion

**Figure 3:**
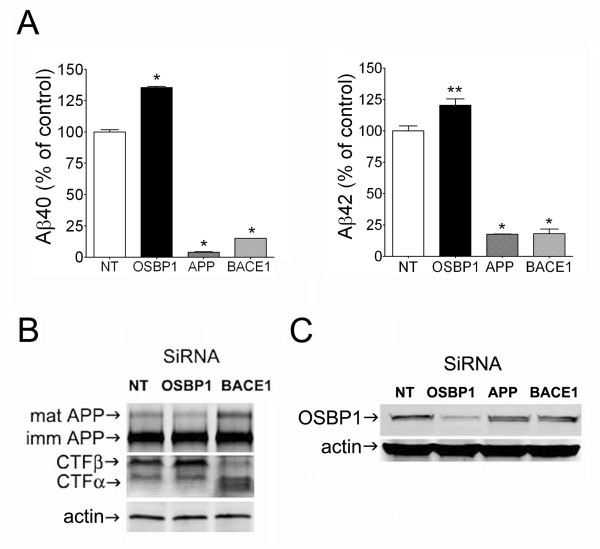
**Knockdown of OSBP1 expression using siRNA increased secreted levels of Aβ40 and Aβ42 and the steady-state levels of CTFβ.** Cells were transfected with the 3 siRNA sequences as described in Methods. Non-targeting siRNA (NT) and siRNA against APP and BACE1 were used as controls. **A**, Media samples were assayed for Aβ40 and Aβ42 by ELISA. **B**, Lysates from HEK-APPNFEV cells were separated by SDS-PAGE and immunoblotted with an antibody to the APP C-terminus for detection of full length APP and APP-CTFs, and to actin as loading control. **C**, Lysates from HEK-APPNFEV cells and immunoblotted with antibodies to OSBP1 and actin to confirm effective knockdown. *p < 0.01 and **p < 0.05 by ANOVA.

To investigate whether knockdown of OSBP1 would result in opposite effects to that of OSBP1 overexpression, three siRNA sequences were designed to specifically target OSBP1. siRNA sequences targeting APP itself and BACE1 were used as positive controls, and media from transfected cells were assayed for Aβ40 and Aβ42 by ELISA. As expected, knockdown of BACE1 or APP markedly decreased Aβ40 and Aβ42 secretion compared to that of non-targeting siRNA control HEK-APPNFEV cells. Conversely to OSBP1 overexpression, knockdown of OSBP1 significantly increased Aβ40 and Aβ42 levels in the media by 35% and 20%, respectively (Figure 3A). The effects of OSBP1 knockdown on APP-CTFβ levels in HEK-APPNFEV cells were consistent with changes in Aβ levels (Figure 3B), i.e., APP-CTFβ was increased when compared to non-targeting siRNA treated cells. While OSBP1 siRNA treatment did not cause significant changes in full-length immature APP (Figure 3B), a decrease in the mature, fully glycosylated APP band was noticed and likely associated with increased APP β-cleavage. However, APP-CTFα was not decreased with OSBP1 knockdown, which is in concert with our previous inconsistent results observed within α-cleaved metabolites when OSBP1 was overexpressed. In contrast to OSBP1, BACE1 siRNA led to increased levels of mature APP, markedly decreased APP-CTFβ and increased APP-CTFα levels (Figure 3B), which are all in agreement with reduced APP β-cleavage. Western blotting of cell lysates confirmed that transfection of OSBP1 siRNA produced effective knockdown of OSBP1 expression in HEK-APPNFEV when compared with non-targeting siRNA pool, or to siRNA pools targeted to APP or BACE1 (Figure 3C). Taken all together, our findings support the hypothesis that OSBP1 overexpression regulates the processing of APP, most likely by modulating its subcellular trafficking.

### Overexpressing OSBP1 modulated the distribution of APP dimers

**Figure 4:**
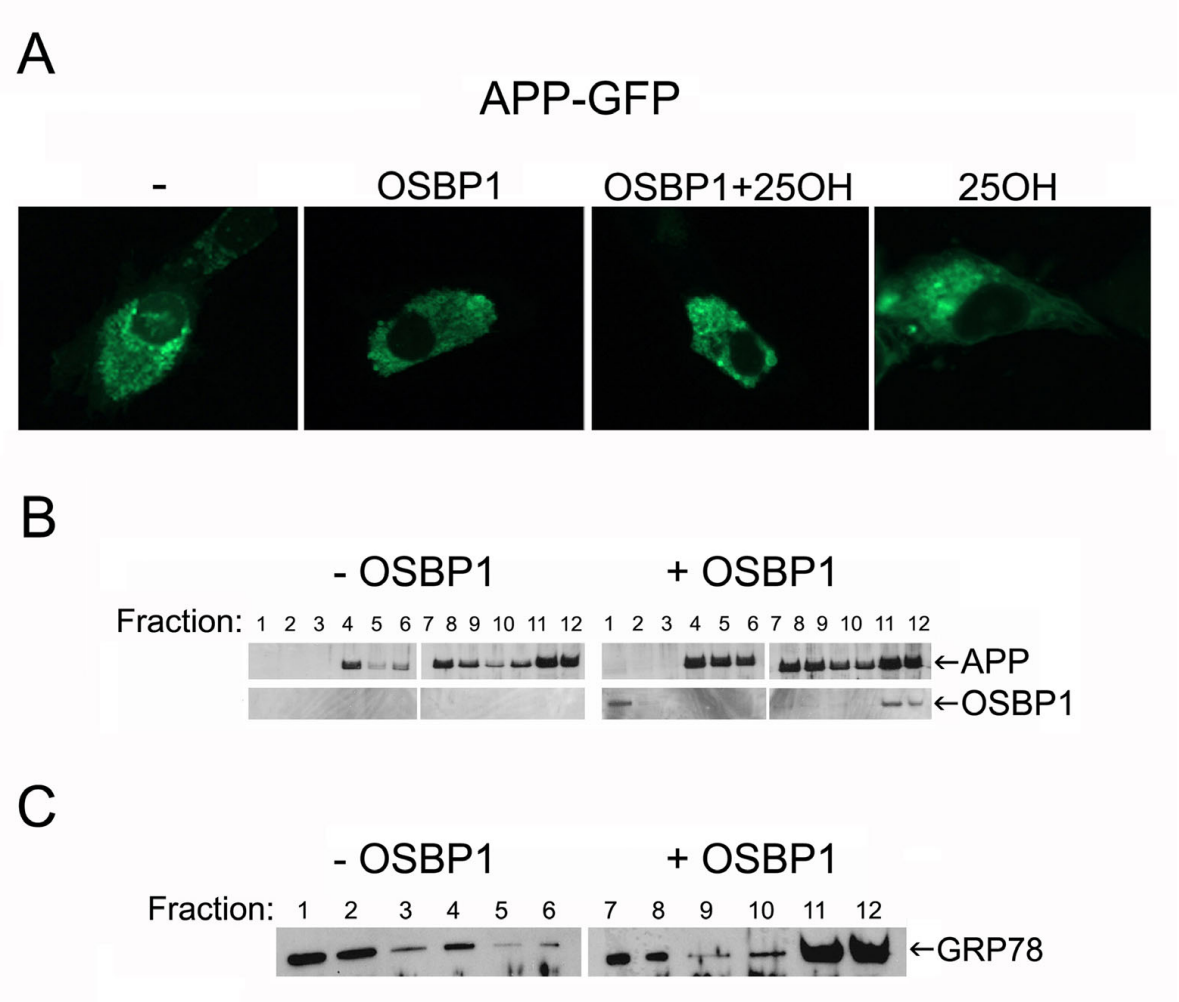
**Subcellular localization of total APP was not affected by OSBP1 overexpression.A**, H4 cells were transfected with GFP-tagged APP with or without OSBP1 and then treated with 1 μM 25OH or vehicle for 24 h. Cells were examined under 40× magnification. **B**, H4 cells overexpressing APP were transfected with vector only (left panel) or OSBP1 (right panel); 48 h later cells were homogenized and separated by sucrose gradient fractionation. Gradient fractions were analyzed by Western blotting using antibodies to the APP C-terminus and to OSBP1. **C**, Fractions were also blotted with an antibody to the ER marker GRP78.

Since no apparent changes in cell surface distribution of APP were detected (Figure 2C), we investigated the potential effects of OSBP1 overexpression on the subcellular localization of APP. Co-expressing OSBP1 with an APP-GFP construct indicated that OSBP1 did not affect the gross intracellular distribution of APP, in the presence or absence of OSBP1 ligand 25OH (Figure 4A). In addition, 25OH treatment did not alter the overall distribution of APP-GFP expressed alone (Figure 4A), and no noticeable changes in the subcellular localization of BACE1-GFP were observed with OSBP1 co-expression (data not shown). We also performed sucrose gradient fractionation of APP-overexpressing cells transiently transfected with OSBP1 or empty vector (Figure 4B and 4C). The majority of OSBP1 coincided with the ER marker GRP78, detected in fractions 11 and 12 (Figure 4B, right panel and 4C). APP was found throughout fractions 4–12, and this distribution was not altered by overexpression of OSBP1 (Figure 4B).

**Figure 5:**
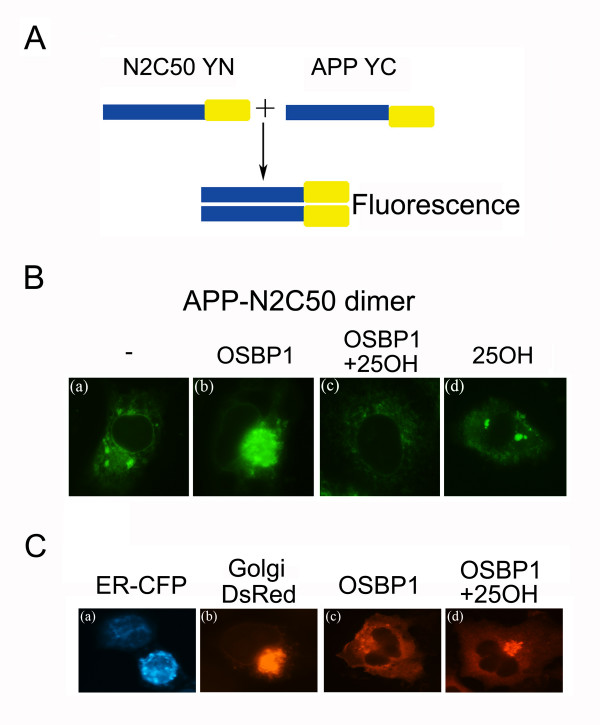
**Localization of APP-N2C50 heterodimers was modulated by OSBP1.A**, Diagram showing the BiFC method. APP and Notch constructs with a C-terminal deletion (N2C50) were fused with the amino or carboxy domains of YFP. Binding of target constructs brings the two halves of YFP together and produces fluorescence. **B**, Cells were transfected with APPYC and N2C50YN with or without OSBP1 and, after 24 h, treated with 1 μM 25OH or vehicle for another 24 h. Detection of the BiFC fluorescence was carried out as described and cells were examined under 63× magnification. **C**, Panels (a) and (b) show fluorescence images of cells expressing P450 2C2/CFP (ER CFP) and 1,4-galactosyltransferase/DsRed2 (Golgi DsRed) markers, acquired 24 h after transfection; panels (c) and (d) show localization of OSBP1 in controls and cells treated for 24 h with 1 μM 25OH, respectively.

We then postulated that OSBP1 might modulate a specific intracellular pool of APP. To investigate this hypothesis we used bimolecular fluorescence complementation (BiFC), by which we have recently been able to identify interactions between APP-APP homodimers and APP-Notch heterodimers [34,35]. Using BiFC we demonstrated that OSBP1 appeared to modulate the distribution of APP-Notch2 heterodimers (Figure 5A). Constructs consisting of Notch2 with a C-terminal deletion fused to the N-terminus of YFP (N2C50YN) and APP fused to the C-terminus of YFP (APPYC) were made as described previously [34]. H4 cells were transfected with APPYC plus N2C50YN in the absence or presence of overexpressed OSBP1 and then treated with 1 μM 25OH or vehicle for 24 h. APPYC plus N2C50YN only yielded BiFC fluorescence showing diffuse staining with a modest amount in a compartment previously shown to be the Golgi apparatus (Figure 5B, panel a). Cells co-transfected with OSBP1 showed almost exclusive fluorescence localization to the Golgi apparatus (Figure 5B, panel b). Treatment of cells overexpressing OSBP1 with 25OH shifted the distribution of APP-N2C50 hetero-dimers to a distribution consistent with ER and plasma membrane localization (Figure 5B, panel c), whereas treatment with 25OH had no effect on heterodimer localization in cells not overexpressing OSBP1 (Figure 5B, panel d). Finally, we correlated the subcellular distribution of OSBP1 to that of the APP-Notch2 heterodimers. Cells were transfected with OSBP1 cDNA, treated with 1 μM 25OH or vehicle for 24 h, fixed, and OSBP1 protein localization was determined by immunofluorescence. OSBP1 labeling exhibited a pattern consistent with cytoplasmic and ER distribution, shifting to exclusively Golgi upon treatment with 25OH (Figure 5C) [36]. Thus, OSBP1 and APP-Notch2 heterodimers localized to non-overlapping compartments and transition of OSBP1 from ER to Golgi induced by 25OH treatment correlated with transition of APP-Notch2 heterodimers from Golgi to ER.

## Discussion

There is increasing evidence from biochemical, epidemiological and genetic findings that cholesterol levels are linked to Aβ production and AD; however, the mechanisms involved are currently poorly understood. Our previous data demonstrate that the catabolic oxidation products of cholesterol, termed oxysterols, potently inhibit secretion of Aβ and sAPP [32]. Oxysterols are known to bind two different targets, LXRs and ORPs. From the three major naturally occurring oxysterols, 27-hydroxycholesterol (27OH), 24(*S*)-hydroxycholesterol (24OH) and 25-hydroxycholesterol (25OH), only 25OH has been reported to bind ORPs. The brain specific 24OH and the peripherally synthesized 27OH have been shown to be primarily LXR ligands [37]. Several studies have examined the effect of LXR agonists on APP processing [19,38,39]. Some of these studies demonstrate that stimulation of LXRs inhibits Aβ production in cell culture and reduces Aβ deposition *in vivo*, although present data in the literature are not entirely consistent [19,38,39]. We hypothesized that 25OH binding to ORPs might be an additional mechanism by which oxysterols modulate APP processing in cells. In support of this hypothesis, the *CH25H *gene encoding for cholesterol 25-hydroxylase and located within the major AD-linkage region of chromosome 10, is ranked third in a list of genes with strongest association with the disease [40]. 25OH-mediated activation of ORPs would likely result in a distinct modulation of APP processing than that of LXRs, since ORPs regulate cholesterol distribution and organelle trafficking, while LXRs are transcription factors which primarily induce genes involved in cholesterol efflux [41,42]. However, it has been recently reported that ORP8 modulates expression levels of the cholesterol-efflux pump ATP-binding cassette A1 (ABCA1) in macrophages [43], and therefore it is possible that ORPs also contribute to the activity of LXRs by modulating cellular levels of LXR-target genes. The present study investigated whether OSBP1, the archetypal member of the family, could alone modulate APP processing.

We found that overexpression of OSBP1 in cells stably expressing APP led to downregulation of the amyloidogenic processing shown by a decrease in APP-CTFβ and sAPPβ levels, as well as Aβ40 and Aβ42 secretion. As expected, knockdown of OSBP1 had the opposite effect in these metabolites. However, the effects of OSBP1 on the non-amyloidogenic processing of APP appear to be more complex. While OSBP1 overexpression increased and OSBP1 knockdown decreased APP-CTFα, sAPPα levels in the media were decreased in OSBP1 overexpressing cells. One possible explanation for these findings is that OSBP1 overexpression also upregulates cellular binding sites for sAPPα, and that this effect is even greater than its effect on APP processing. sAPPa has neuroprotective and neurotrophic functions and it is several orders of magnitude more potent than sAPPβ because it contains a heparin-binding domain at the C-terminus, enabling binding to several proteoglycans involved in cell adhesion, cell-cell, or cell-matrix interactions, cell growth, and synaptic plasticity [44,45]. Therefore, OSBP1 upregulation could be beneficial by both decreasing the amyloidogenic processing of APP and favoring the neuroprotective actions of sAPPα.

OSBP1 and other members of the ORP family have previously been implicated in the control of intracellular trafficking. For example, ORP2 has been shown to enhance endocytosis [41] and many other family members including OSBP1 are known to be involved in vesicular transport [29,46,47]. In addition, OSBP1 itself can traffic between the ER/cytoplasm and the Golgi depending on whether it is bound to cholesterol or 25OH. OSBP1 overexpression did not appear to produce changes in APP subcellular localization. However, by using bimolecular fluorescence complementation to study the movement of APP dimers, we found that OSBP1 overexpression caused sequestration of APP-Notch2 dimers in the Golgi. Addition of 25OH, which induces translocation of OSBP1 from the ER/cytoplasm to the Golgi, caused APP-Notch2 dimers to translocate from the Golgi to the ER. When OSBP1 was not overexpressed, 25OH had no effect on the localization of the APP-Notch2 dimers, perhaps because basal levels of OSBP1 were not high enough to counteract the effects of 25OH binding to other ORPs or LXRs. These results suggest that OSBP1 might act to exclude a specific population of APP heterodimers from organelles containing high levels of OSBP1, which is likely governed by changes in subcellular cholesterol distribution.

It is of interest that while OSBP1 overexpression produced no obvious changes in the localization of APP, an altered localization of APP-Notch2 dimers was detected. Interaction between APP and Notch2 was recently described and shown to occur in the ER, Golgi and at the plasma membrane [34,35]. However, the importance of this interaction is currently unclear. In addition to binding to Notch2, APP is known to bind to many other proteins and therefore other types of APP heterodimers can potentially exist. For example, SorL1, a ~250 kDa receptor that has recently been shown to have genetic association with AD [48], is a potential trafficking partner to APP. Overexpression of SorL1 has similar effects on APP processing to that of OSBP1, including decreased in β-site cleavage and shift in subcellular APP localization from ER and plasma membrane to Golgi and early endosomes [49]. These effects were shown to occur via direct interaction between SorL1 and APP. SorL1 has also been shown to interact with BACE1 and it appears to compete with APP for interaction with BACE1 in the Golgi [50]. It is possible that a similar mechanism may exist whereby APP and OSBP1 both compete for binding to SorL1 or other similar trafficking proteins.

In this particular study our investigation was limited to APP-Notch2 heterodimers; however, this is likely only a small proportion of the total population of APP heterodimers, and it is possible that OSBP1 could also affect the intracellular trafficking of other types of APP heterodimers. The homodimerization of APP has been previously suggested to positively regulate Aβ production [51,52]. Our present results indicate that the localization of APP-Notch2 dimers could also influence the processing of APP, and therefore suggests that APP heterodimers may play an important role in the regulation of APP trafficking and Aβ generation.

## Conclusion

The present findings provide the first evidence for an involvement of oxysterol binding proteins in the regulation of APP processing/trafficking. OSBP1 binds oxysterols as well as cholesterol, and therefore this work also provides further insight into the relationship between cholesterol and APP metabolism. We hypothesize that OSBP1 plays a pivotal role in APP metabolism when cellular cholesterol levels are increased. In this model, an increase in cellular cholesterol causes OSBP1 to shift to cytoplasm/ER while APP-Notch dimers localize to the Golgi, and Aβ production remains low. However, in cells expressing low levels of OSBP1, an increase in cholesterol levels could not be accommodated by such mechanism allowing for Aβ levels to increase, as it has been shown in previous studies examining the effects of cholesterol on Aβ production [53,54]. Many questions regarding these relationships still remain to be answered and more work will be required to determine whether high levels of OSBP1 do indeed protect against the detrimental effects of increased cellular cholesterol. If this were the case, OSBP1 and/or other ORPs may prove to be promising targets in the search for novel AD therapeutic.

## Methods

### Generation of constructs

The pcDNA3.1 plasmid containing myc-His tagged rabbit OSBP1 was provided by Dr. R.G. Anderson (University of Texas, TX). The plasmid encoding the ER marker (P450 2C2 CFP) was kindly provided by Dr. Elzbieta Skorupa (UIUC, Urbana, IL) [55]. The plasmid encoding the Golgi marker (81 N-terminal amino acids of human 1,4-galactosyltransferase/DsRed2) was kindly provided by Dr. Nikolaj Klocker (University of Freiburg, Germany) [56]. Human OSBP1 cDNA was obtained from Invitrogen and cloned into 6.2 cLumio-DEST vector (Invitrogen, Carlsbad, CA).

### Cell culture

H4 human neuroglioma cells stably overexpressing APP (H4-APP) were provided by Dr. T. Golde (Mayo Clinic, Jacksonville, FL). Cells were cultured in OptiMEM containing FBS (10%), non-essential amino acids, penicillin (50 U/ml), streptomycin (50 μg/ml) and zeocin (100 μg/ml). Transient transfections were carried out when cells were 60–70% confluent using FuGENE (Roche, Indianapolis, IN) according to the manufacturer's instructions. Treatment with 25OH (1 μM) was carried out for 24 h, beginning 24 h after transient transfection. HEK293T stably overexpressing APPNFEV cells were generated as previously described [33]. APPNFEV encodes human APP isoform 1–695, modified at amino acid positions 595–598 by substitution of the amino acid sequence KMDA for the NFEV sequence. Cells were maintained in DMEM containing 10% FBS, penicillin (100 U/ml), streptomycin (100 μg/ml) and puromycin (2 μg/ml). HEK-APPNFEV cells (40–50% confluence) were transfected with the OSBP1 SiRNA pool including the following sequences: GAUAGAUCAGUCUGGCGAATT; CUUUGAGCUGGACCGAUUATT; GAGAAUACUGGGAGUGUAATT; or non-targeting SiRNA control pool (Dharmacon, Lafayette, CO) at a final concentration of 300 nM siRNA using Oligofectamine (Invitrogen, Carlsbad, CA) according to the manufacturer's instructions. For overexpression experiments, the human OSBP1 cDNA was transfected into HEK-APPNFEV cells (70–80% confluence) using Lipofectamine 2000 (Invitrogen, Carlsbad, CA) according to the manufacturer's instructions. After overnight incubation, transfection mixture was removed from each well and replaced with 100 μl of DMEM containing 10% FBS. Fresh medium was conditioned for 24 h before collection for analysis of APP metabolites by ELISA. Cell viability was assessed at the end of each experiment using the AlamarBlue reagent (Biosource, Carlsbad, CA).

### Aβ ELISA

Cell-secreted Aβ levels were detected in the conditioned media by sandwich ELISA. 50 μl of 20-fold diluted media plus 50 μl of alkaline phosphatase (AP)-conjugated G210 (for Aβ40 detection), 50 μl of 2-fold diluted media plus 50 μl of 12F4 (for Aβ42 detection) or 50 μl of 100-fold diluted media plus 50 μl of P21 (for sAPPα detection) were incubated overnight on ELISA plates coated with 6E10. CPD-star (Tropix, Bedford, MA) was used as AP substrate for detection using the Analyst AD microplate reader (Molecular Devices, Sunnyvale, CA).

### SDS-PAGE and Western blot analysis

H4-APP cells were lysed 48 h after transfection in 20 mM Tris containing 1% Triton X-100 and a protease inhibitor cocktail (Sigma, St. Louis, MO). Proteins were separated by SDS-PAGE and then immunoblotted using antibodies to the C-terminus of APP (Calbiochem, San Diego, CA), c-myc (Sigma, St. Louis, MO) and actin (Sigma, St. Louis, MO). Cells for sAPPα analysis were transfected with OSBP1 and after 48 h, the medium was removed and cells were treated with 1 μM phorbol 12-myristate 13-acetate (PMA) for 30 min in fresh serum-free medium. The media were collected and concentrated using a Vivaspin column (Vivascience, Hannover, Germany). Media samples were immunoblotted with the 6E10 antibody to detect sAPPα (Signet Labs, Cambridge, MA), or the 192 wt antibody to sAPPβ, which was provided by Dr. Peter Seubert (Elan Pharmaceuticals, CA). Densitometric analysis of blots was carried out using the Un-Scan-It automated digitizing system. For evaluation of OSBP1, APP, and APP-CTFs expression levels in HEK-APPNFEV, cell monolayers were washed twice with ice-cold PBS and lysed in PBS containing 1% Triton X-100, 1 mM PMSF and Complete protease inhibitor cocktail (Roche, Indianapolis, IN) for 30 min on ice. Cell lysates were cleared of cell debris by centrifugation at 14,000 rpm for 3 min at 4°C. Supernatants were analyzed for protein assay and equal amounts of total cell protein were separated by PAGE. Samples were separated in 4–15% or 7.5% Tris-glycine gels for OSBP1 and APP or 16% Tris-tricine gels for APP-CTFs analysis. Proteins were transferred to PVDF membranes and probed with anti-OSBP1 goat polyclonal (ABCAM, Cambridge, MA), anti-β-APP rabbit polyclonal (Zymed, Carlsbad, CA) or anti-actin mouse monoclonal (Sigma, St. Louis, MO) antibodies. Blots were developed using ECL Plus (Amersham Biosciences, Piscataway, NJ) and scanned with the Typhoon variable mode imager (Amersham Biosciences, Piscataway, NJ).

### Cell surface biotinylation

Cell surface biotinylation experiments were performed using the Cell Surface Protein Isolation kit from Pierce (Pierce, Rockford, IL). Briefly, HEK-APPNFEV cells were plated in 10 cm dishes and transfected with OSBP1 cDNA as described above. 48 h following transfection, cell monolayers were quickly rinsed twice with ice-cold PBS and biotinylation of cell surface proteins was carried our according to manufacturer instructions. After quenching of unbound biotin, cells were scrapped and incubated on ice for 30 min in 500 μl of lysis buffer. An aliquot (20 μl) of clarified supernatant was reserved for running total cell protein gels and the remaining sample was applied to immobilized NeutrAvidin™ gel columns for separation of biotin-labeled cell surface proteins eluted in 200 μl sample buffer. Western blots were carried out as above.

### Sucrose gradient fractionation

H4-APP cells were homogenized 48 h after transfection in 10 mM Tris (pH 7.4) containing 0.25 M sucrose, 1 mM MgAc_2 _and a protease inhibitor cocktail. The homogenate was loaded on top of a step gradient consisting of 1 ml of 2 M sucrose, 4 ml of 1.3 M sucrose, 3.5 ml of 1.16 M sucrose and 2 ml of 0.8 M sucrose (all in 10 mM Tris, pH 7.4 containing 1 mM MgAc_2_). The gradient was centrifuged at 100,000 g for 2.5 h. Twelve 1 ml fractions were then collected from the top of the gradients and analyzed by SDS-PAGE and Western blotting using antibodies to the C-terminus of APP, OSBP1 or GRP78 (Santa Cruz Biotechnology, Santa Cruz, CA).

### Bimolecular fluorescence complementation (BiFC)

The preparation of the APPYC and N2C50YN constructs was described previously [34]. Wild type H4 cells were grown on a Lab-Tek II 8-chambered coverglass (Nalge Nunc, Rochester, NY) and co-transfected with the expression vectors indicated in each experiment using FuGENE reagent (Roche, Indianapolis, IN). 24 to 48 h after transfection, cells were rinsed twice with PBS and resultant fluorescence was observed in living cells using a Zeiss Axiovert 200 M microscope with YFP, CFP and rhodamine filters. OSBP1 was detected using anti c-myc antibody, followed by the Alexa Fluor 594 goat anti-mouse IgG antibody (Molecular Probes, Eugene, OR) under 63× magnification.

## Abbreviations

The abbreviations used are: ADAM, a disintegrin and metalloprotease; TACE, TNFα-converting enzyme; BACE1, β-site APP-cleaving enzyme; NSAIDs, nonsteroidal anti-inflammatory drugs; PMA, phorbol 12-myristate 13-acetate.

## Competing interests

CVZ, SS, WJR and GRS are full-time employees of Merck and Co., Inc. and declare financial competing interests. JC, CC, MG, CRA and BW have no competing interests.

## Authors' contributions

CVZ and JMC designed and carried out APP processing experiments, performed statistical analysis and draft the manuscript; CC and MG performed the bimolecular fluorescence experiments; SS carried out APP processing experiments, WJR helped conceive of the study; GRS helped conceive of the study and draft the manuscript; CRA participated in design and analysis of bimolecular fluorescence experiments; BW conceived of the study, oversaw experiments, analyzed data and edited the manuscript. All authors read and approved of the manuscript.
